# *RAI14* in the blood feather regulates chicken pigmentation

**DOI:** 10.5194/aab-63-231-2020

**Published:** 2020-07-14

**Authors:** Adeyinka Abiola Adetula, Xiaolei Liu, Liubin Yang, Chengchi Fang, Hui Yu, Hua Li, Shijun Li

**Affiliations:** 1 Key Laboratory of Agricultural Animal Genetics, Breeding, and Reproduction, Ministry of Education, College of Animal Science and Veterinary Medicine, Huazhong Agricultural University, Wuhan, China; 2 Genome Analysis Laboratory of the Ministry of Agriculture and Rural Affairs, Agricultural Genomic Institute at Shenzhen, Chinese Academy of Agricultural Sciences, Shenzhen, China; 3 Lingnan Guangdong Laboratory of Modern Agriculture, Shenzhen, China; 4 College of Life Science, Foshan University, Foshan, Guangdong, China

## Abstract

A genome-wide association study (GWAS) was performed on a resource family consisting of white and colored chickens for identification of genes related to plumage coloration using the Fixed and random model Circulating Probability Unification (FarmCPU) package. GWAS identified three chromosomal single-nucleotide polymorphisms (SNPs), demonstrating the polygenic basis of plumage phenotypes. Herein, retinoic acid-induced protein 14 (*RAI14*), a developmentally regulated gene that encodes a protein containing many ankyrin repeats, was identified as a candidate gene involved in plumage color. In this study, mRNA expression profiles of chicken *RAI14* were determined, indel (insertion–deletion) variants were identified, and their association was analyzed in white and colored chickens. *RA114* mRNA was expressed in all tissues tested (brain, spleen, liver, heart, oviduct, kidney, lung, pituitary gland, ovary, muscle, feather bulb, and skin). A relatively high *RAI14* expression in white feather bulb compared to colored feather bulb (P<0.01) indicated a potential association with plumage color. Additionally, statistical analysis revealed that a 4 bp indel genetic variation in *RAI14* was associated with plumage phenotypes (P<0.01). Together, our analysis of the identification of the *RAI14* gene will enable us to understand the genetic mechanisms behind chicken pigmentation.

## 1 Introduction

Color is an important feature of the majority of living organisms. In poultry, complete white is characterized by a uniform, pure white color across all feathers, which is generally associated with no visible pigment, while colored chickens are mainly characterized by the distribution of two melanin pigments: eumelanin (black, gray, and brown colors) and pheomelanin (reddish-brown colors). Previous studies reported that six nucleotide deletions in the tyrosinase (*TYR*) gene is associated with albino white (Tobita-Teramoto et al., 2000), and Chang reported that retroviral insertion of the avian leukosis virus in the fourth intron of the *TYR* gene was associated with white recessive (Chang et al., 2006). Genetic studies have also shown that mutations in the premelanosome protein (*PMEL17*) gene have been associated with dominant white (Kerje et al., 2004), and five mutations in the solute carrier family 45 member 2 (*SLC45A2*) have been associated with the silver color in chicken (Gunnarsson et al., 2007). Extended black, dark brown, and sex-linked barring patterns were associated with mutations in the coding region of the melanocortin receptor 1 (*MC1R*) gene (Kerje et al., 2003). The dark brown plumage is caused by a 8.3 kb deletion upstream of *SOX10* (Gunnarsson et al., 2011), and sex-linked barring is controlled by the cyclin-dependent kinase inhibitor 2A/B (*CDKN2A/B*) locus (Hellström et al., 2010), suggesting that nucleotide polymorphism plays a significant role in differentiation that underlies plumage color in chickens.

Currently, genome-wide association studies (GWASs) have a potential role to play in poultry breeding (Xie et al., 2012; Nie et al., 2016; Pértille et al., 2017). As a result, the current study integrated a GWAS to estimate the association effects of single-nucleotide polymorphisms (SNPs) on plumage phenotypes using a 600 k SNP marker panel (Kranis et al., 2013). From the GWAS analysis results, significant associations were found within chromosome 1 (Chr. 1), Chr. 7, and Chr. Z. In the present study, we have focused on the Z chromosome due to strong associations that include SNP linked to *RAI14*, a gene involved in plumage coloring.

Chicken *RAI14* is an active metabolite of vitamin A in the retinoid family. Through nuclear receptors, retinoids have potent effects on cell differentiation, proliferation, and apoptosis (Ross et al., 2000), and their pathways have often been suggested in reviews to contain genetic factors that regulate gene expression (Blomhoff and Blomhoff, 2006; Bastien and Rochette-Egly, 2004; McGrane, 2007). *RAI14* reacts through the classical pathway regulated by retinoic acid, where its expression is induced by all-trans retinoic acid (ATRA). Studies on human melanocytes to investigate the melanogenic activity of ATRA based on spectrophotometric measurement of melanin content and enzymatic tyrosinase activity have shown that RA-induced genes are capable of significantly increasing melanin and tyrosinase contents (Baldea et al., 2013). To date, chicken *RAI14* gene expression profiles and DNA polymorphisms have remained unexplored. Therefore, this study examined the tissue expression profiles of the *RAI14* gene, identified a novel 4 bp mutation, and identified the relationship between the base pair mutation and plumage phenotype in white and colored chickens. Our findings provide a basis for further investigation into the underlying causal mutation and suggest hypotheses for further study.

## 2 Materials and methods

### 2.1 Ethics statement

The protocols for all animal experiments were conducted under a project authorization approved by the Scientific Ethics Committee of the Huazhong Agricultural University with approval number HZAUCH-2015-005.

### 2.2 Experimental birds and sample collection

Commercial chickens were obtained from the poultry farm of Huadu Yukou Poultry Industry Co. Ltd, Beijing, China. Briefly, one rooster with black plumage was mated with one Yukou Brown I hen to generate the F1 (filial 1) individuals. Using the parental and F1 individuals, two different F1 intercross (F2) and one backcross populations were designed to be segregated. More than 1000 individuals were generated, and a total of 384 individuals were selected for genome-wide association study. The phenotype score system is based on the proportion of white (i.e., completely white) and colored (i.e., brown and black) chickens. According to the phenotype score, 384 chickens were divided into two basic phenotypes: white and colored. In total, 125 individuals were classified as representatives of the white group and the remaining chickens as the colored group.

### 2.3 Discovery of functional genes via genome-wide association study

Genomic DNA of 384 chickens was extracted from the blood samples using the TIANamp Blood DNA Kit (Tiangen Biotech Co. Ltd., Beijing, China). The purity and concentration of genomic DNA were measured using a NanoVue spectrophotometer (Harvard Bioscience, Inc, USA). DNA quality and concentration of each sample were calculated, and equal amounts of DNA were sent to a commercial genotyping company (Shanghai Bohao Biotechnology Co., Ltd., Shanghai, China) using a 600 k Affymetrix^®^ Axiom^®^ HD genotyping array (Kranis et al., 2013). In order to increase the accuracy of the GWAS analysis, PLINK software was used to apply strict quality control criteria including (1) animal call rate >95 % and call frequency >95 %; (2) minor allele frequency (MAF) threshold set at 0.03; and (3) SNPs that strongly deviated from Hardy–Weinberg equilibrium ($P<10^{-6}$) (Purcell et al., 2007). Overall, the qualified chickens (371) and SNPs (436 861) were retained for further statistical analyses. For population structure, a stratification approach was used using a mixed model that included fixed effects (overall mean and covariates) and random effects (SNP effect, individual effect, and residual errors), as implemented in FarmCPU software (Liu et al., 2016a). In the current study, all chickens were raised in the same individual cages; therefore, only the first principal component was used as a covariate to account for population structure in the analysis. Furthermore, based on the location of the phenotype-associated SNPs, the candidate gene regions were thus determined upstream and downstream of the associated SNPs (100 kb). Therefore, candidate genes were been searched for within these defined regions using Ensembl from BioMart (Kinsella et al., 2011). The National Center for Biotechnology Information (NCBI) database (https://www.ncbi.nlm.nih.gov/gene/, last access: 13 July 2020) was used to search for gene annotations and functions in chickens and related animals.

**Figure 1:**
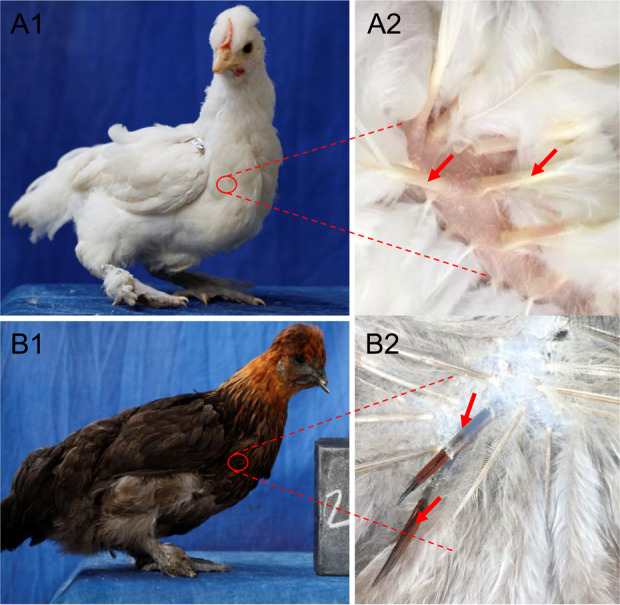
Examples of plumage in chicken: **(a1)** and **(a2)** are white; **(b1)** and **(b2)** are colored. Red arrows indicate feather bulbs.

### 2.4 Validation experiment

#### 2.4.1 RNA extraction, cDNA synthesis, and RT-PCR assay

Total RNA was extracted from white and colored feather bulbs (Fig. 1) using TRIzol reagent (Invitrogen, CA, USA) according to the manufacturer instructions with some modifications. The RNA quality and quantity were rated by 260 nm to 280 nm and 260 nm to 230 nm ratios using a NanoDrop 2000 spectrophotometer (Thermo Scientific™ ND2000USCAN, Waltham, MA USA). According to the NanoDrop measurements, all RNA samples with optical density (OD) 260/280 ratio of 1.9–2.0 and OD 260/230 ratio of >1.8 were reverse transcribed to cDNA using PrimeScript™ RT reagent kit (Takara Biotechnology Co. Ltd.) according to manufacturer recommendations. The 25 µL qPCR reaction contained 12.5 µL 2× SYBR qPCR mix (Aidlab Biotechnologies Co., Ltd., China), 0.5 µL (10 µM) of each primer, and 1 µL cDNA (Adeyinka et al., 2017). Polymerase chain reaction (PCR) amplification was carried out using a Bio-Rad Laboratories, Inc. device at 95 ${}^{\circ }$C for 2 min, followed by 40 cycles at 95 ${}^{\circ }$C for 10 s and 60 ${}^{\circ }$C for 15 s. The qPCR amplifications were conducted using an independent set of 10 biological and 3 technical replicates per sample. Relative quantification of *RAI14* gene expression was evaluated by the 2${}^{-\Delta \Delta \mathrm{Ct}}$ methods (Livak and Schmittgen, 2001) using the chicken beta-actin as a reference gene. Data were subjected to statistical analysis using GraphPad Prism 5 software (Motulsky, 2007). P<0.05 was considered the threshold of statistical significance. Next, we examine *RAI14* expression in different tissues: 2 µg total RNA from 12 tissues including brain, spleen, liver, heart, oviduct, kidney, lung, pituitary gland, ovary, muscle, feather bulb, and skin were digested with RNase-free DNase I (Cat #RRO47A China) to eliminate genomic DNA. The isolated RNA was used for first-strand cDNA synthesis with PrimeScript™ RT reagent kit (Cat #RRO47B) in 20 µL reactions, as indicated by the manufacturers. RT-PCR was employed to examine *RAI14* tissue expression using RNAs from different tissues (including brain, spleen, liver, heart, oviduct, kidney, lung, pituitary gland, ovary, muscle, feather bulb, and skin). RT-PCR was performed in a 25 µL reaction which contained 12.5 µL 2× SYBR qPCR mix (Aidlab Biotechnologies Co., Ltd., China), 0.5 µL (10 µM) of each primer, and 1 µL cDNA. The reaction conditions comprised an initial denaturation at 94 ${}^{\circ }$C for 3 min, followed by 32 cycles of 94 ${}^{\circ }$C for 30 s, annealing at 60 ${}^{\circ }$C for 20 s, 72 ${}^{\circ }$C for 20 s, and a final extension at 72 ${}^{\circ }$C for 5 min. Beta-actin gene was used as a control gene. The primer pairs used in this study are shown in Table 1.

**Table 1:** Primers used for indel locus detection and expression analysis.

Primer (NC_006127.4)	Primer sequence (5${}^{\prime }$–3${}^{\prime }$)	Length (bp)	Function
*RAI14*-X1, X2, X3, X4-F	GGAACGTCAGATTGTCACAG	152	qPCR
*RAI14*-X1, X2, X3, X4-R	GAATGGTGATCCTGAGAAAGT		
*RAI14*-X5-F	GACGACGACGACGACCACGA	225	qPCR
*RAI14*-X5-R	ACTTTCTCAGGATCACCATT		
*RAI14*-X7-F	TTGCAAGTGATGTAGGCGT	187	qPCR
*RAI14*-X7-R	ACTTTCTCAGGATCACCATT		
*RAI14*-X8-F	CACCACCACCACCACCGGAG	204	RT-PCR & qPCR
*RAI14*-X8-F	ACTTTCTCAGGATCACCATT		
Beta-Actin-F	GAGAGAGAAATTGTGCGTGA	212	qPCR
Beta-Actin-R	ATGATGGAGTTGAAGGTAGT		
*RAI14*-NC_006127.4-4bp-1F	GCTCATTCTTGCAGGAGAGG	1710	Indel detection
*RAI14*-NC_006127.4-4bp-1R	AATGACAAATGTGCTCGGAC		
*RAI14*-NC_006127.4-4bp-2F	TACCAGTTATTTAAATGAGA	95	Indel genotyping
*RAI14*-NC_006127.4-4bp-2R	AAGTGCAGTTAGTTGAATAG		

**Figure 2:**
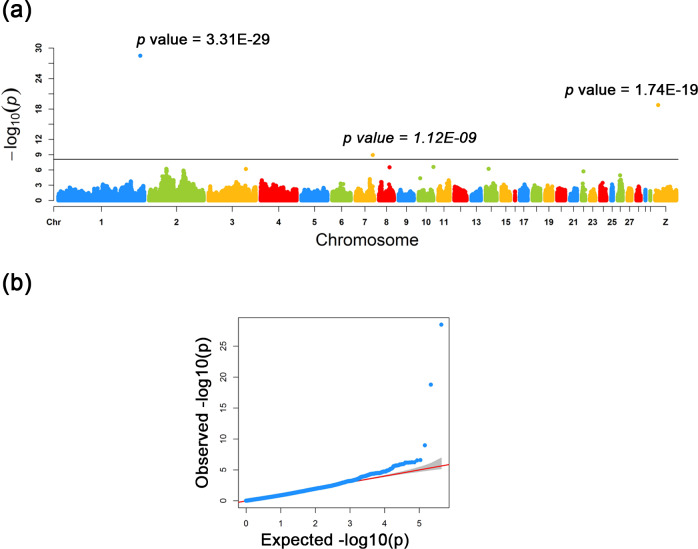
Manhattan and quantile–quantile plots for the three loci identified across the chicken genome. **(a)** The negative log10 P values of SNPs transformed from a genome-wide scan were plotted against positions on the chromosomes under FarmCPU models. The three significant associated SNPs surpassed the Bonferroni threshold (horizontal gray lines) for the plumage trait using the FarmCPU method. **(b)** The quantile–quantile (QQ) plot assessed the negative base 10 logarithms of the P value observed on the Y axis, and the X axis is the expected negative base 10 logarithms of the P values under the assumption that the P values follow a uniform [0, 1] distribution.

**Figure 3:**
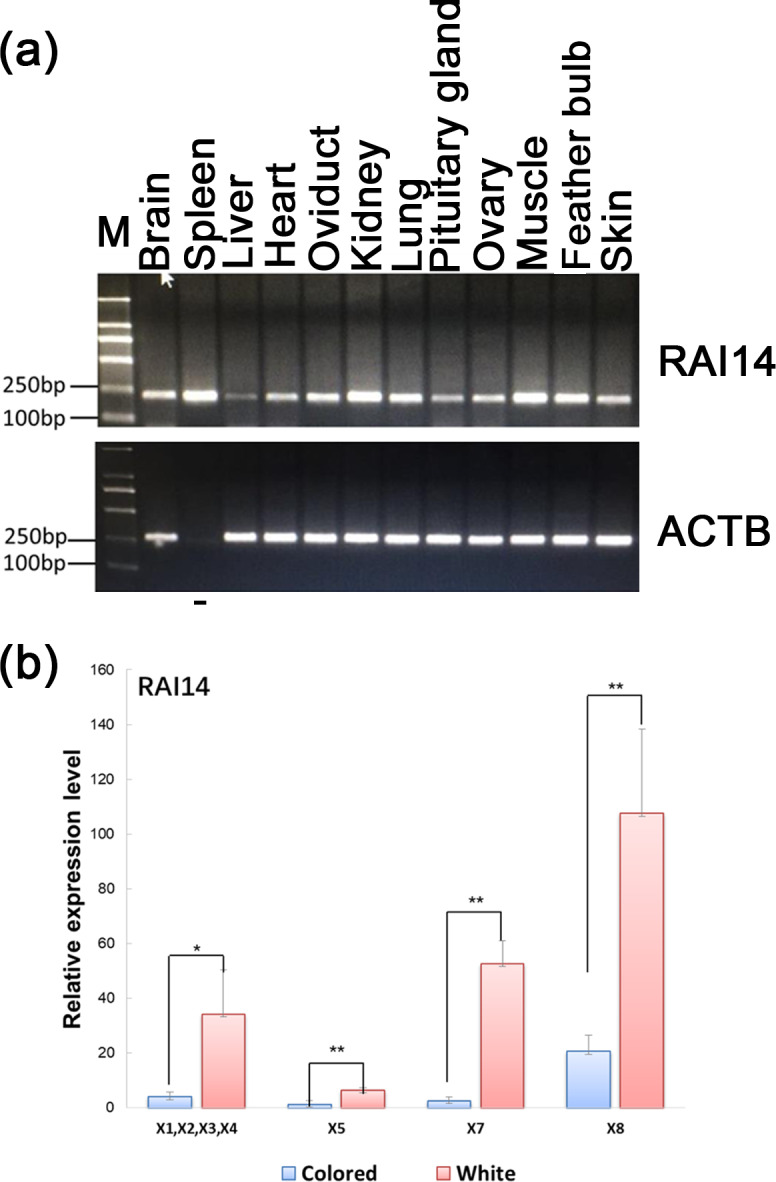
Expression of *RAI14* mRNA detected in feather bulbs and different tissues. **(a)** *RAI14* mRNA expression profiles in different tissues by RT-PCR. Lengths of the PCR products (*RAI14*: 204 bp; beta-actin: 212 bp) (-), no cDNA samples are marked in the figure; M represent molecular weight (MW): 2000 bp. **(b)** Expression of *RAI14* mRNA in the feather bulb by qPCR. All samples were normalized by beta-actin. Each bar represents the relative expression level of the *RAI14* gene mRNA. Error bars represent standard error of the mean (SE). X1, X2, X3, X4, X5, X7, and X8 denote RAI14 mRNA isoforms. ${}^{*}$ represents significant at p<0.05 and ${}^{**}$ represents significant at p<0.01, based on assuming unequal variances and Student's t test.

#### 2.4.2 Sequencing, indel identification, and genotyping

Chicken genomic DNA was extracted from blood samples using a standard phenol–chloroform methods (Sambrook and Russell, 2006). The quality and quantity of all DNA samples were measured with 1 % agarose gel electrophoresis and a NanoDrop™ ND-2000c spectrophotometer (Thermo Scientific, Inc.). DNA samples of 30 chickens were selected randomly to construct a DNA pool with equal DNA concentration of 50 ng µL${}^{-1}$ for each of white and colored phenotypes. An indel (insertion–deletion) mutation was identified using pairs of primers based on the chicken *RAI14* gene (GenBank accession no. NC_006127.4). The primer sequences used for detecting indel mutation are listed in Table 1. PCR amplifications were performed in a 25 µL volume containing 50 ng pooled DNA, 2.5 µL of 10× PCR buffer, 5 mM of dNTPs, 10 pmol of forward and reverse primer, 0.625 U Taq DNA polymerase (Takara Biotechnology Co. Ltd.), and double-distilled water (ddH2O). The reactions were performed under the following conditions: an initial pre-denaturing at 95 ${}^{\circ }$C for 5 min; 35 cycles of 95 ${}^{\circ }$C (20 s), 55–60 ${}^{\circ }$C (20 s), and 72 ${}^{\circ }$C (20 s); and a final step at 72 ${}^{\circ }$C for 5 min. The PCR products were sequenced directly with an ABI3730xl DNA analyzer (Applied Biosystems), and indel discovery was conducted by the Chromas software (version 2.3.1) and DNAMAN (version 6.0). The PAGE-based (Polyacrylamide gel electrophoresis) genotyping assay was utilized for genotyping of the 4 bp indel in 57 white and 52 colored individuals. A 20 bp DNA ladder (Dye Plus, code no. 3420A) was used for the gel electrophoresis. The acrylamide gel was cast on washed plates with 1.5 mm spacer. The annealed PCR products (primers listed in Table 1) were resolved by electrophoresis in non-denaturing polyacrylamide gels containing 30 % acrylamide–bisacrylamide (29:1, w/w), 1× tris–borate–EDTA (TBE), ammonium persulfate, and tetramethylethylenediamine (TEMED). After 8 h of electrophoresis at 80 V and 200 mA, polyacrylamide gel was immersed in a sodium hydroxide + sodium carbonate anhydrous solution for 10 min and silver nitrate reagent solution for 7–8 min before visualization.

#### 2.4.3 Genetic parameters and association analysis

The genotype and allele frequencies of the 4 bp indel in *RAI14* were calculated using the SHEsis program (http://analysis.bio-x.cn, last access: 13 July 2020) (Li et al., 2009). Methods developed by Nei and Roychoudhury (1974) were used to calculate population genetic diversity indices, including homozygosity (Ho) and heterozygosity (He; Ho + He = 1), where He and Ho are measures of genetic variation of a population. The chi-square ($\chi^{2}$) test was used to evaluate the Hardy–Weinberg equilibrium (HWE) (Pouresmaeili et al., 2013). The statistical significance level was set at P<0.05. For each genotype, association analysis was carried out using a linear model implemented in SAS (SAS v. 9.2, SAS Institute Inc., Cary, NC, USA). Briefly, the linear model can be generally specified as

1 $$Y_{ijkl}=\mu +B_{i}+G_{j}+A_{k}+e_{ijkl},$$

where $Y_{ijkl}$ is the individual observation for a trait, μ is population mean, $B_{i}$ is the effect of breed, $G_{j}$ is the effect of genotype, $A_{k}$ is the effect of age, and $e_{ijkl}$ is random error. Differences in means were considered significant at P<0.05. The least square means (LS means) were estimated with standard errors for different genotypes and plumage traits. Age, breed, and genotypes were considered fixed effects, and plumage traits were dependent variables. The SAS PROC TTEST procedures (SAS v. 9.2, SAS Institute Inc., Cary, NC, USA) were used to test the differences in the LS means between genotypes.

**Figure 4:**
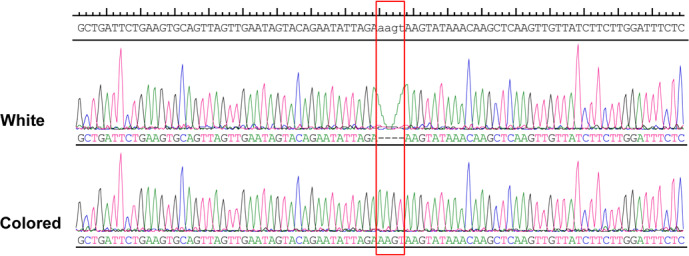
Sequence chromatograms of genomic regions harboring the indel. Red highlighted portion indicates insertion–deletion in the white and colored animals.

## 3 Results

### 3.1 Identification of candidate genes for plumage trait

As a result of a GWAS conducted for white and colored chickens using FarmCPU methods (Liu et al., 2016b), three quantitative trait loci (QTLs) at a genome-wide significant level were found to be associated with the plumage traits (Fig. 2). We also identified three significant associated SNPs, including rs75377751:A > G, rs77034318:T > G and rs77188285:T > G that surpassed the Bonferroni threshold ($p<10^{-8}$) (Fig. 2 and Table 2). All SNPs had a MAF of above 20 %. The strongest associated SNP had a P value of $3.31\times 10^{-29}$. Among the three significantly associated SNPs (rs75377751:A > G, rs77034318:T > G and rs77188285:T > G) located on Chr. 1, Chr. 7, and Chr. Z (Table 2), SNP rs75377751:A > G was found to be located directly within the QTL region defined in previous studies and stated to be responsible for the white trait in chicken (Sato et al., 2007). Next, the chicken genome assembly (Gallus_gallus-5.0; GCA_000002315.3) was used to identify candidate genes within the defined regions for the associated SNPs. All three associated SNPs had hits directly on candidate genes, including *TYR* (Sato et al., 2007), *RAI14*, and glycosyltransferase-like domain-containing protein 1 (*GTDC1*) (Table 2). Nevertheless, there is a possibility for the discovery of candidate genes around these related SNPs with expanded knowledge of gene function.

**Table 2:** Location, frequency, and P values of significantly associated SNPs from the genome-wide association study.

SNP (position)	Chr.	MAF	P value	Candidate gene (position)
rs75377751 (187920668)	1	0.245283	$3.31\times 10^{-29}$	*TYR* (187920666…187970514)
rs77034318 (33305561)	7	0.309973	$1.12\times 10^{-9}$	*GTDC1* (33270007…33450697)
rs77188285 (10474311)	Z	0.474394	$1.74\times 10^{-19}$	*RAI14* (10453674…10546697)

The location is indicated by the position of the chromosome and base pair. The frequency is indicated by the MAF (minor allele frequency).

### 3.2 Profile of chicken *RAI14* in feather bulb and various tissues

Investigation of the general tissue expression of *RAI14* was explored by RT-PCR, using beta-actin as an internal control. *RAI14* was expressed in all 12 tissues, with the highest level in brain, spleen, oviduct, kidney, lung, muscle, feather bulb, and skin, while its expression in liver, heart, pituitary gland, and ovary was relatively weak (Fig. 3a). We further observed *RAI14* expression in feather bulb by qPCR. As shown in Fig. 3b, the expression levels of *RAI14* mRNA isoforms (X1, X2, X3, X4, X5, X7, and X8) were significantly higher in white than in colored chickens. According to these findings, white and colored chickens differed significantly at P<0.01 and P<0.05 levels. Previous experiments with chick retinal pigment epithelial cells indicated that retinoic acid inhibits cell proliferation and induces melanin synthesis (Kishi et al., 2001). Together, these results indicate that *RAI14* has a potentially important function to play in plumage coloring. Next, to explore possible DNA markers that account for this variability in expression, we concentrated on the detection of polymorphisms in *RAI14*.

### 3.3 Genetic variation of *RAI14* gene in white and colored chicken

More than 4 kb upstream of the *RAI14* gene sequence was analyzed. These analyses identified a number of potential candidate mutations, among which a 4 bp candidate indel was detected by DNA sequencing (NC_006127.4, Chr. Z: 10 457 847–10 457 850). The 4 bp indel (AAGT) was genotyped for subsequent association study in 57 white and 52 colored chickens. The PCR products used in the study showed the sequence diagrams of the novel 4 bp indel (Fig. 4).

**Table 3:** Genetic parameters and association analysis of the 4 bp indel of the *RAI14* gene.

	Frequencies		Genetic parameters			
Indel locus	Genotypes	Alleles	Ho	He	HWE (P value*)	Association p value${}^{c}$
4 bp (n=52)${}^{a}$	II (44, 0.846)	0.923 (I)	0.858	0.142	0.361 (0.548)	$2.20\times 10^{-16}$
	ID (8, 0.154)	0.077 (D)				
	DD (0, 0.000)					
4 bp (n=57)${}^{b}$	II (8, 0.140)	0.237 (I)	0.639	0.362	12.386 (0.0004)	
	ID (11, 0.193)	0.763 (D)				
	DD (38, 0.667)					

${}^{a}$ colored; ${}^{b}$ white; HWE: Hardy–Weinberg equilibrium; Ho: homozygosity; He: heterozygosity; ${}^{c}$ HWE p values were computed using the chi-square test.

### 3.4 Associations between indel variations and plumage traits

The genetic parameters associated with the *RAI14* indel locus were determined (Table 3); the results indicated that insertion allele “I” (0.923) of the 4 bp indel was often seen than deletion allele “D” (0.077) in the colored chickens. For the white chickens, the 4 bp indel frequency of the I allele was lower (0.237), and for the D allele it was higher (0.763), Table 3. In addition, the chi-square test showed that the frequency of the 4 bp indel genotype was in accordance with HWE (P>0.05) in colored chickens. However, the 4 bp indel did not agree with HWE (P<0.05; Table 3) in white chickens. Next, the associations between the *RAI14* 4 bp indel locus and plumage trait were investigated. The findings showed that there was a relationship between the 4 bp indel and the plumage trait (P<0.0001; Table 3).

## 4 Discussion

In order to explore the genetic basis of variation in plumage phenotypes, we reported GWAS in an F2 population consisting of white and colored chickens. Due to the small sample size and population structure, strict analyzes were used to control stratification using FarmCPU models (Liu et al., 2016b). Finally, a total of three QTLs with three underlying SNPs were found to be significantly associated with the plumage phenotypes on Chr. 1, Chr. 7, and Chr. Z. We performed candidate gene searches within the QTL regions, and one of the identified candidate genes was supported by QTL studies on white plumage (*TYR* locus, Chr. 1), although the previous studies measured white trait differently and in different breeds (Sato et al., 2007). The overlap of one of our candidate genes for white plumage in chicken also provided hints that genes related to white trait may be shared across species. Meanwhile, we found no direct evidence linking *GTDC1* gene on Chr. 7 to tyrosinase enzymatic activity.

Interestingly, on Chr. Z, we found a new candidate gene (*RAI14* locus); previous studies have shown that retinoic acid-induced genes inhibit growth and enhance the differentiation of melanoma cells in vitro (Huang et al., 2003). Retinoic acid (RA) is a morphogen derived from retinol (vitamin A) that plays a vital role in cell growth, differentiation, and organogenesis (Kam et al., 2012), indicating that *RAI14* may be a key regulator of biological functions in chicken development and pigment cell differentiation. Therefore, to determine the role of the *RAI14* gene in plumage coloring, we identified the regulatory variants affecting *RAI14* gene expression in order to better understand the mechanisms that regulate the chicken pigment system. In addition, there are no previous reports of *RAI14* chicken tissue expression profiles. The relationship between *RAI14* gene variants and plumage traits in the white and colored populations required further investigation.

First, we determined the tissue expression profiles of the chicken *RAI14* gene, and the results showed widespread distribution of *RAI14* in the brain, spleen, liver, heart, oviduct, kidney, lung, pituitary gland, ovary, muscle, feather bulb, and skin; these results suggested that *RAI14* has a broad and significant function in various tissues. We further determined its expression patterns in the white and colored chicken feather bulb. Interestingly, our results showed that *RAI14* expression levels in white chickens were higher when compared to colored chickens (P<0.05 and P<0.01), suggesting *RAI14* may be associated with plumage variation. In addition, previous reports indicated that the *RAI14* gene regulates retinal pigment epithelium, which provides nutrients to photoreceptor cells and contains melanin pigments that absorb excess light radiation (Kutty et al., 2001). Together, these data indicated that the *RAI14* gene could play an important role in pigmentation.

**Figure 5:**
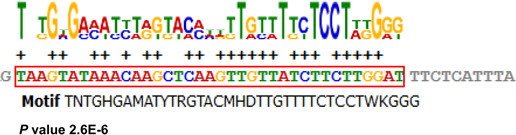
Bioinformatics predicts transcription factor binding sites on the 4 bp indel sequences. One potential transcriptional factor *FOXC1* (Forkhead box C1) appears only at insertion sequence. Red highlights the *FOXC1* factor binding site sequence.

DNA sequence analysis of white and colored chickens revealed genetic variants of *RAI14*, including a 4 bp indel, and statistical analysis showed that the 4 bp locus was in HWE (P>0.05) in colored chickens (Table 3). In addition, there was a significant association between the 4 bp indel variations and the plumage color (Table 3). Previous investigations, however, have shown that retinoic acid-stimulated genes are capable of regulating the transcription of a number of genes by their ability to bind specific nuclear receptors, i.e., retinoic acid receptors and retinoid X receptors (Gudas, 1994a; Hofmann, 1994), indicating that the *RAI14* gene variant is capable of transcribing and regulating hundreds of genes by mediating their effect and binding to specific DNA sequences (Gudas, 1994b). The transcription factor binding site to the 4 bp indel was therefore predicted using the JASPAR database, version 2014 (http://jaspar2014.genereg.net/, last access: 13 July 2020). Depending on the transcription factor binding site prediction results, Forkhead box C1 (*FOXC1*), a transcription factor, may be bound in the presence of insertion of 4 bp nucleotides (Fig. 5). This discovery made it possible for *FOXC1* to influence chicken pigmentation. However, *FOXC1* is a DNA-binding transcriptional factor that plays a role in a wide range of cellular and developmental processes (Saleem et al., 2004; Berry et al., 2002); of course, the question of whether the *RAI14* 4 bp indel influences plumage phenotype by linking it to the *FOXC1* locus needs further study.

## 5 Conclusions

In conclusion, we identified three chromosomal SNPs that were significantly associated with plumage coloration by a GWAS. The novel candidate gene *RAI14* mRNA was expressed in all tissues tested (brain, spleen, liver, heart, oviduct, kidney, lung, pituitary gland, ovary, muscle, feather bulb, and skin), and the expression levels in the feather bulbs were significantly different between white and colored chickens. A novel 4 bp indel in the *RAI14* promoter region was also identified as a candidate mutation related to the plumage phenotype. These findings would provide valuable resources for future studies to improve understanding of the genetic architecture of plumage coloring and, eventually, to improve marker-assisted selection (MAS) in chicken breeding.

## Data Availability

The data sets are available upon request from the corresponding author.
